# Predictors of use of direct oral anticoagulants in patients with venous thromboembolism: Findings from the Registro Informatizado Enfermedad Tromboembólica registry

**DOI:** 10.3389/fmed.2022.991376

**Published:** 2022-11-25

**Authors:** Alicia Lorenzo, Patricia Beroiz, Salvador Ortiz, Jorge del Toro, Lucia Mazzolai, Alessandra Bura-Riviere, Adriana Visonà, Peter Verhamme, Pierpaolo Di Micco, Giuseppe Camporese, Teresa Sancho Bueso, Manuel Monreal, the RIETE Investigators

**Affiliations:** ^1^Department of Internal Medicine, Hospital Universitario La Paz, Madrid, Spain; ^2^Department of Geriatrics, Hospital Germans Trias i Pujol, Badalona, Barcelona, Spain; ^3^Department of Medicine, Universidad Autónoma de Barcelona, Barcelona, Spain; ^4^Department of Applied Economics, Universidad Autónoma Madrid, S&H Medical Science Service Advisor, Madrid, Spain; ^5^Department of Internal Medicine, Hospital General Universitario Gregorio Marañón, Madrid, Spain; ^6^Department of Angiology, Centre Hospitalier Universitaire Vaudois (CHUV), Lausanne, Switzerland; ^7^Department of Vascular Medicine, Hôpital de Rangueil, Toulouse, France; ^8^Department of Vascular Medicine, Ospedale Castelfranco Veneto, Castelfranco Veneto, Italy; ^9^Vascular Medicine and Haemostasis, University of Leuven, Leuven, Belgium; ^10^Department of Internal Medicine and Emergency Room, Ospedale Buon Consiglio Fatebenefratelli, Naples, Italy; ^11^Angiology Unit, Department of Cardiac, Thoracic and Vascular Sciences, Padua University Hospital, Padua, Italy; ^12^Department of Internal Medicine, Hospital Germans Trias i Pujol, Badalona, Barcelona, Spain; ^13^Chair for the Study of Thromboembolic Disease, Faculty of Health Sciences, UCAM—Universidad Católica San Antonio de Murcia, Murcia, Spain

**Keywords:** venous thromboembolism, direct oral anticoagulants, anticoagulant therapy, predictors, RIETE, different countries

## Abstract

**Background:**

Current guidelines recommend the use of direct oral anticoagulants (DOACs) for patients with venous thromboembolism (VTE). However little is known about the use of DOACs in daily practice.

**Methods:**

We used the RIETE registry to identify predictors of use of DOACs for initial and/or long-term therapy of VTE based on patient-related factors, institution-related factors or over time.

**Results:**

Among 41,678 patients from March 2013 to September 2021, 12,286 (29%) used DOACs: for initial therapy 6,456; for long-term therapy 12,046. On multivariable analysis, independent predictors were: age < 65 years (odds ratio [OR]: 1.30; 95% CI: 1.23–1.38), body weight <50 kg (OR: 0.54; 95% CI: 0.45–0.65) or >120 kg (OR: 0.64; 95% CI: 0.53–0.77), initial VTE presentation as pulmonary embolism (OR: 1.18; 95% CI: 1.13–1.25), recent bleeding (OR: 0.53; 95% CI: 0.45–0.63), renal insufficiency (OR: 0.44; 95% CI: 0.38–0.51), liver cirrhosis (OR: 0.32; 95% CI: 0.20–0.52), thrombocytopenia (OR: 0.40; 95% CI: 0.34–0.49), atrial fibrillation (OR: 1.58; 95% CI: 1.42–1.75) and prior VTE (OR: 1.14; 95% CI: 1.06–1.22). The DOACs were more likely used in other European countries (OR: 8.97; 95% CI: 8.49–9.49), America (OR: 6.35; 95% CI: 5.67–7.11) or in other countries of the world (OR: 2.99; 95% CI: 2.70–3.31) than in Spain, and progressively increased from 2013–2015 to 2016–2018 (OR: 2.78; 95% CI: 2.62–2.95) and 2019–2021 (OR: 6.36; 95% CI: 5.95–6.80).

**Conclusion:**

In this large multinational VTE registry, variations were observed in the use of DOACs according to patient or country factors, and over time. The safety, costs, and influence of the DOACs on VTE-related outcomes in daily practice warrant further investigation.

## Introduction

Current guidelines of anticoagulant therapy recommend the use of direct oral anticoagulants (DOACs) for initial and long-term therapy of patients with venous thromboembolism (VTE) (1, 2). The risk reduction of recurrent VTE with DOACs is similar to the risk reduction with low-molecular-weight heparin (LMWH) and vitamin K antagonists (VKAs), while the risk of bleeding is less with DOACs than with standard therapy (3). However, the use of DOACs has not completely replaced the use of standard therapy. There are patient-related factors, and also institutional or logistical reasons that may limit the use of DOACs in daily practice. Patient-related factors include older age, extreme body weights (where there may be doubts about the optimal dose) or concomitant diseases (where there may be concern about the risk of bleeding) (4–12). In addition, resource availability may also drive the choice of therapies. A better knowledge of the reasons why physicians prescribe the use of DOACs for the initial and/or long-term therapy of VTE could lead to design randomized trials for subgroups of patients where its use is lower than expected (to reassure on the efficacy and safety of DOACs) or higher than expected (to avoid undesirable outcomes).

The RIETE (Registro Informatizado Enfermedad TromboEmbólica) registry is an international, ongoing registry of consecutive patients with symptomatic, objectively confirmed, acute VTE (ClinicalTrials.gov identifier: NCT02832245). Since its inception in 2001, data from this registry have been used to evaluate outcomes after acute VTE, such as the frequency of recurrent VTE, bleeding and mortality, and risk factors for these outcomes (13). In the current study, we aimed to determine the potential variations in the use of DOACs in patients with confirmed VTE, based on patient-related factors, institution-related factors, and over time.

## Patients and methods

### Inclusion criteria

Consecutive patients with acute deep vein thrombosis (DVT) or pulmonary embolism (PE) confirmed by objective tests (compression ultrasonography for suspected DVT; helical CT-scan, ventilation-perfusion lung scintigraphy or conventional angiography for suspected PE) were enrolled in RIETE. Patients were excluded if they were currently participating in a therapeutic clinical trial with a blinded therapy. All patients (or their legal power of attorney) provided written or oral consent for participation in the registry, in accordance with local ethics committee requirements.

### Study design

Data were collected from March 2013 (corresponding to the time when the prescription of DOACs was allowed) to July 2021. The primary goal of this study was to determine the potential variations in the use of DOACs in patients with symptomatic, objectively confirmed VTE, based on patient-related factors, or institution-related factors. As such, the main outcome was the proportion of patients using DOACs vs. those using other anticoagulant drugs. Secondary outcomes were the proportion of patients using each DOAC (vs. the other DOACs), and the proportion of patients using lower-than recommended doses of DOACs. Recommended dosing was defined as dosing consistent with FDA-labeled dosing for treatment of VTE as of September 2021.

Patient-related factors explored in this study included demographics (sex, age, body weight), initial VTE presentation (PE with or without concomitant DVT vs. isolated DVT), concomitant diseases that could contraindicate the use of DOACs [including recent (<30 days before) major bleeding, biopsy-proven liver cirrhosis, creatinine clearance (CrCl) levels <30 mL/min and platelet count <100,000/μLat baseline], and concomitant disorders that could lead to prolong the duration of anticoagulant therapy (prior VTE and atrial fibrillation). We also evaluated the proportion of fragile patients that used DOACs (fragile patients defined as those aged ≥75 years, with CrCl levels ≤50 mL/min or body weight ≤50 kg) (14). Institutional factors assessed in the current study included the country of enrolment. Further, we explored the trends in the use of DOACs over the study years.

### Treatment

Patients were managed according to the clinical practice of each participating hospital (i.e., there was no standardization of treatment). The decision on the type and duration of therapy was left to the attending physicians. Patients were followed-up for at least 3 months in the outpatient clinic or physician’s office.

### Statistical analysis

Categorical variables were compared using the chi-square test (two-sided) and Fisher’s Exact Test (two-sided). Continuous variables were compared using Student *t* test. To identify predictors of prescription of drugs we used logistic regression analyses. All the analyses were adjusted for sex, age (65 years; 65–79; >79 years), body weight (<50 kg; 50–120; >120 kg), initial VTE presentation (symptomatic PE; isolated DVT), recent major bleeding, liver cirrhosis, CrCl levels at baseline <30 mL/min, platelet count <100,000/μL, atrial fibrillation, prior VTE, the country where the VTE was diagnosed (Spain; other European countries; America; rest of the world) and years of VTE diagnosis (2013–2015; 2016–2018; 2019–2021). Odds ratios (OR) and corresponding 95% confidence intervals (CI) were calculated, and a *p* value < 0.05 was considered to be statistically significant. Statistical analyses were conducted with SPSS for Windows Release 25.0 (SPSS, Inc.).

### Role of the funding source

The sponsors of the RIETE registry (Sanofi, Leo Pharma and Rovi) had no role in study design, data collection, data analysis, data interpretation or writing of the report. The corresponding author had full access to all the data in the study and had final responsibility for the decision to submit for publication.

## Results

Among 41,678 patients with VTE recruited from January 2013 to September 2021 in RIETE, 12,286 (29%) used DOACs: 6,456 for initial therapy and 12,046 for long-term therapy. Among the 41,678 patients, 20,896 (50%) were men; mean age was 65 ± 17 years; 23,458 (56%) initially presented with PE; 970 (2.3%) had recent major bleeding; CrCl levels <30 mL/min 2,097 (5.0%); liver cirrhosis 209 (0.5%); platelet count < 100,000/μL 1,055 (2.5%); atrial fibrillation 2,361 (5.7%) and prior VTE 5,818 (14%). In total, 16,767 patients (40%) were fragile.

**FIGURE 1:**
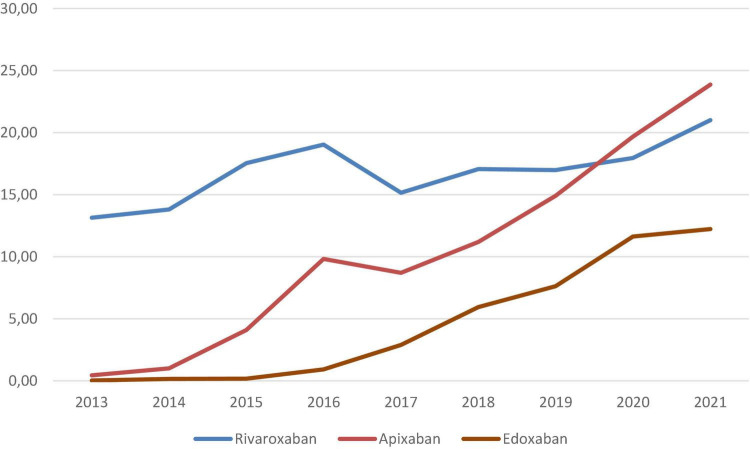
Cumulative rates of patients receiving DOACs for initial and/or long-term therapy of VTE over time.

Most patients (65%) were attended in Spanish centers, 25% in other European countries, 3.7% in America and 6.0% in the rest of the world (Table 1). For initial therapy, 5,141 patients (12%) used rivaroxaban and 1,315 (3.2%) apixaban. For long-term therapy, 6,631 patients (16%) used rivaroxaban, apixaban 3,548 (8.5%), edoxaban 1,473 (3.5%), and dabigatran 394 (0.9%). The proportion of patients using DOACs progressively increased over time (Figure 1).

**TABLE 1:** Prescription of DOACs over time in different countries.

	Total	2013–2014	2015–2016	2017–2018	2019–2021
**All patients**	***41,678***	***10,526***	***10,798***	***10,653***	***9,701***
Rivaroxaban initially	5,141 (12.3%)	1,041 (9.9%)	1,481 (13.7%)	1,288 (12.1%)	1,331 (13.7%)
Apixaban initially	1,315 (3.2%)^‡^	5 (0.1%)^‡^	226 (2.1%)^‡^	402 (3.8%)^‡^	682 (7.0%)^‡^
Rivaroxaban long-term	6,631 (15.9%)	1,367 (13.0%)	1,929 (17.9%)	1,658 (15.6%)	1,677 (17.3%)
Apixaban long-term	3,548 (8.5%)^‡^	74 (0.7%)^‡^	727 (6.7%)^‡^	1,025 (9.6%)^‡^	1,722 (17.7%)
Edoxaban long-term	1,473 (3.5%)^‡^	9 (0.1%)^‡^	54 (0.5%)^‡^	451 (4.2%)^‡^	959 (9.9%)^‡^
Dabigatran long-term	394 (0.9%)^‡^	21 (0.2%)^‡^	111 (1.0%)^‡^	140 (1.3%)^‡^	122 (1.3%)^‡^
**Spain**	***27,245***	***6,542***	***7,066***	***7,122***	***6,515***
Rivaroxaban initially	991 (3.6%)	198 (3.0%)	228 (3.2%)	256 (3.6%)	309 (4.7%)
Apixaban initially	341 (1.2%)^‡^	**1 (0.02%)** ^‡^	43 (0.6%)^‡^	76 (1.1%)^‡^	221 (3.4%)^‡^
Rivaroxaban long-term	1,879 (6.9%)	**326 (5.0%)**	460 (6.5%)	498 (7.0%)	595 (9.1%)
Apixaban long-term	1,659 (6.1%)^‡^	**53 (0.8%)** ^‡^	305 (4.3%)^‡^	437 (6.1%)*	864 (13.3%)^‡^
Edoxaban long-term	873 (3.2%)^‡^	8 (0.1%)^‡^	32 (0.4%)^‡^	184 (2.6%)^‡^	649 (10.0%)
**Europe, other**	***10,414***	***2,647***	***2,873***	***2,419***	***2,475***
Rivaroxaban initially	3,308 (31.8%)	624 (23.6%)	1,068 (37.2%)	794 (32.8%)	822 (33.2%)
Apixaban initially	730 (7.0%)^‡^	2 (0.1%)^‡^	156 (5.4%)^‡^	264 (10.9%)^‡^	308 (12.4%)^‡^
Rivaroxaban long-term	3,795 (36.4%)	793 (30.0%)	1,232 (42.9%)	901 (37.2%)	869 (35.1%)
Apixaban long-term	1,488 (14.3%)^‡^	10 (0.4%)^‡^	351 (12.2%)^‡^	484 (20.0%)^‡^	643 (26.0%)^‡^
Edoxaban long-term	586 (5.6%)^‡^	0	21 (0.7%)^‡^	266 (11.0%)^‡^	299 (12.1%)^‡^
**America**	***1,530***	***446***	***294***	***543***	***247***
Rivaroxaban initially	454 (29.7%)	165 (37.0%)	118 (40.1%)	85 (15.6%)	86 (34.8%)
Apixaban initially	116 (7.6%)^‡^	1 (0.2%)^‡^	9 (3.1%)^‡^	33 (6.1%)^‡^	73 (29.5%)
Rivaroxaban long-term	549 (35.9%)	190 (42.6%)	153 (52.0%)	112 (20.6%)	94 (38.1%)
Apixaban long-term	169 (11.0%)^‡^	3 (0.7%)^‡^	17 (5.8%)^‡^	60 (11.1%)^‡^	89 (36.0%)
Edoxaban long-term	2 (0.1%)^‡^	0	1 (0.3%)^‡^	1 (0.2%)^‡^	0
**Rest of the world**	***2,489***	***891***	***565***	***569***	***464***
Rivaroxaban initially	388 (15.6%)	54 (6.1%)	67 (11.9%)	153 (26.9%)	114 (24.6%)
Apixaban initially	128 (5.1%)^‡^	1 (0.1%)^‡^	18 (3.2%)^‡^	29 (5.1%)^‡^	80 (17.2%)*
Rivaroxaban long-term	408 (16.4%)	58 (6.5%)	84 (14.9%)	147 (25.8%)	119 (25.6%)
Apixaban long-term	232 (9.3%)^‡^	8 (0.9%)^‡^	54 (9.6%)*	44 (7.7%)^‡^	126 (27.2%)
Edoxaban long-term	12 (0.5%)^‡^	1 (0.1%)^‡^	0	0	11 (2.4%)^‡^

Differences between patients receiving rivaroxaban vs. other drugs: *p < 0.05; ^†^*p* < 0.01; ^‡^*p* < 0.001. Bold words are the main subjects to study. Years of prescription, number of patients Italic words apply for the total number of patients in each period of study and in the different parts of the world.

### Predictors of use of direct oral anticoagulants vs. other drugs

Overall, 12,286 patients (29%) used DOACs for initial and/or for the long-term therapy of VTE. The proportion of patients using DOACs was highest among those aged <65 years (33%), with atrial fibrillation (33%) or prior VTE (34%), and lowest in patients with CrCl levels < 30 mL/min (15%), liver cirrhosis (11%) or thrombocytopenia (16%) (Table 2) The use of DOACs was lowest in Spain (17%) and highest in other European countries (59%) or America (50%), and progressively increased over time: from 17% in 2013–2015 to 47% in 2019–2021. Among 12,286 patients using DOACs, 2,154 (18%) used lower-than recommended doses. The subgroups of patients that were more likely to use lower-than recommended doses of DOACs were: patients aged >79 years (30%), with CrCl levels <30 mL/min (44%) or with atrial fibrillation (30%). Only 4,105 of the 17,767 fragile patients with VTE (24%) used DOACs: 25% at lower than recommended doses.

**TABLE 2:** Univariable and multivariable analyses for predictors of use of DOACs vs. other drugs.

	Total patient	Patients with DOACs	Patients with DOACs at low doses	Any DOACs (vs. other drugs)	
				
				Univariable OR (95% CI)	Multivariable OR (95% CI)
***Patients, N***	***41,678***	***12,286 (29%)***	***2,154 (18%)***		
Male gender	20,896	6,323 (30%)	1,011 (16%)	1.08 (1.03–1.12)*	1.01 (0.96–1.06)
Age 65–79 years	14,110	3,957 (28%)	627 (16%)	Ref.	Ref.
Age < 65 years	17,944	6,009 (33%)	825 (14%)	1.29 (1.23–1.36)^‡^	1.30 (1.23–1.38)^‡^
Age > 79 years	9,624	2,320 (24%)	702 (30%)	0.81 (0.77–0.86)^‡^	0.96 (0.89–1.03)
Body weight 50–120 kg	39,960	11,882 (30%)	2,073 (17%)	Ref.	Ref.
Body weight < 50 kg	977	190 (19%)	54 (28%)	0.57 (0.49–0.67)^‡^	0.54 (0.45–0.65)^‡^
Body weight > 120 kg	740	213 (29%)	27 (13%)	0.96 (0.81–1.12)	0.64 (0.53–0.77)^‡^
**Initial VTE presentation**					
Isolated DVT	18,220	5,020 (27%)	904 (18%)	Ref.	Ref.
Pulmonary embolism	23,458	7,266 (31%)	1,250 (17%)	1.18 (1.13–1.23)^†^	1.18 (1.13–1.25)^‡^
**Concomitant disorders**					
Recent major bleeding	970	224 (23%)	57 (25%)	0.71 (0.61–0.83)^†^	0.53 (0.45–0.63)^‡^
CrCl levels < 30 mL/min	2,097	310 (15%)	137 (44%)	0.40 (0.35–0.45)^†^	0.44 (0.38–0.51)^‡^
Liver cirrhosis	209	23 (11%)	6 (26%)	0.29 (0.19–0.45)^†^	0.32 (0.20–0.52)^‡^
Platelet count < 100,000/μL	1,055	168 (16%)	46 (27%)	0.45 (0.38–0.53)^†^	0.40 (0.34–0.49)^‡^
Atrial fibrillation	2,361	783 (33%)	237 (30%)	1.20 (1.10–1.31)^†^	1.58 (1.42–1.75)^‡^
Prior VTE	5,818	1,994 (34%)	357 (18%)	1.30 (1.22–1.37)^†^	1.14 (1.06–1.22)^‡^
Fragile patients	16,767	4,105 (24%)	1,034 (25%)	0.66 (0.63–0.69)^†^	–
**Countries**					
Spain	27,245	4,645 (17%)	859 (18%)	Ref.	Ref.
Rest of Europe	10,414	6,152 (59%)	956 (16%)	7.02 (6.68–7.38)^‡^	8.97 (8.49–9.49)^‡^
America	1,530	768 (50%)	130 (17%)	4.90 (4.41–5.45)^‡^	6.35 (5.67–7.11)^‡^
Rest of the world	2,489	721 (29%)	209 (29%)	1.98 (1.81–2.18)^‡^	2.99 (2.70–3.31)^‡^
**Years**					
2013–2015	16,114	2,784 (17%)	502 (18%)	Ref.	Ref.
2016–2018	15,863	4,954 (31%)	968 (20%)	2.17 (2.06–2.29)^‡^	2.78 (2.62–2.95)^‡^
2019–2021	9,701	4,548 (47%)	684 (15%)	4.23 (3.99–4.47)^‡^	6.36 (5.95–6.80)^‡^

*p < 0.05; ^†^*p* < 0.01; ^‡^*p* < 0.001. DOACs, direct oral anticoagulants; CI, confidence intervals; VTE, venous thromboembolism; DVT, deep vein thrombosis; CrCl, creatinine clearance; Ref., reference. Bold words are the main subjects to study. Years of prescription, number of patients Italic words apply for the total number of patients in each period of study and in the different parts of the world.

On multivariable analysis, independent predictors for the use of DOACs (vs. other anticoagulants) were: age <65 years (OR: 1.30; 95% CI: 1.23–1.38) body weight <50 kg (OR: 0.54; 95% CI: 0.45–0.65) or > 120 kg (OR: 0.64; 95% CI: 0.53–0.77) initial VTE presentation as PE (OR: 1.18; 95% CI: 1.13–1.25) recent bleeding (OR: 0.53; 95% CI: 0.45–0.63) CrCl levels <30 mL/min (OR: 0.44; 95% CI: 0.38–0.51) liver cirrhosis (OR: 0.32; 95% CI: 0.20–0.52) platelet count <100,000/μL (OR: 0.40; 95% CI: 0.34–0.49) atrial fibrillation (OR: 1.58; 95% CI: 1.42–1.75) and prior VTE (OR: 1.14; 95% CI: 1.06–1.22) (Table 3) The use of DOACs was more likely in other European countries (OR: 8.97; 95% CI: 8.49–9.49) America (OR: 6.35; 95% CI: 5.67–7.11) or in the rest of the world (OR: 2.99; 95% CI: 2.70–3.31) than in Spain, and progressively increased from 2013–2015 to 2016–2018 (OR: 2.78; 95% CI: 2.62–2.95) and 2019–2021 (OR: 6.36; 95% CI: 5.95–6.80).

**TABLE 3:** Multivariable analyses for predictors of use of every DOAC (vs. the rest of DOACs).

	Rivaroxaban	Apixaban	Edoxaban
***Patients, N***	***6,886***	***3,601***	***1,473***
**Clinical characteristics**			
Male gender	1.20 (1.10–1.30)^‡^	0.88 (0.81–0.95)^†^	0.89 (0.79–1.00)
Age 65–79 years	Ref.	Ref.	Ref.
Age < 65 years	1.47 (1.34–1.61)^‡^	0.72 (0.65–0.79)^‡^	0.88 (0.77–1.01)
Age > 79 years	0.70 (0.62–0.78)^‡^	1.46 (1.30–1.64)^‡^	0.99 (0.84–1.17)
Body weight 50–100 kg	Ref.	Ref.	Ref.
Body weight < 50 kg	1.04 (0.75–1.43)	1.06 (0.77–1.47)	0.91 (0.56–1.50)
Body weight > 120 kg	1.21 (0.88–1.67)	1.04 (0.75–1.44)	0.53 (0.29–0.97)*
**Initial VTE presentation**			
Isolated DVT	Ref.	Ref.	Ref.
Pulmonary embolism	1.16 (1.07–1.26)^‡^	1.08 (0.99–1.17)	0.64 (0.57–0.72)^‡^
**Concomitant disorders**			
Recent major bleeding	0.58 (0.43–0.79)^‡^	1.77 (1.33–2.36)^‡^	0.88 (0.54–1.43)
CrCl levels < 30 mL/min	0.88 (0.67–1.14)	1.10 (0.85–1.41)	1.17 (0.82–1.67)
Liver cirrhosis	1.23 (0.48–3.19)	0.24 (0.05–1.05)	3.86 (1.21–12.3)*
Platelet count < 100,000/μL	0.93 (0.66–1.30)	1.18 (0.84–1.65)	0.84 (0.48–1.48)
Atrial fibrillation	0.72 (0.61–0.85)^‡^	1.22 (1.04–1.44)*	0.81 (0.63–1.05)
Prior VTE	1.03 (0.92–1.14)	1.10 (0.99–1.23)	0.80 (0.67–0.95)*
**Countries**			
Spain	Ref.	Ref.	Ref.
Rest of Europe	2.17 (1.99–2.36)^‡^	0.67 (0.61–0.73)^‡^	0.56 (0.50–0.63)^‡^
America	2.80 (2.32–3.37)^‡^	0.82 (0.68–0.98)*	0.02 (0.00–0.06)^‡^
Rest of the world	2.67 (2.24–3.18)^‡^	0.97 (0.82–1.16)	0.07 (0.04–0.12)^‡^
**Years**			
2013–2015	Ref.	Ref.	Ref.
2016–2018	0.20 (0.17–0.22)^‡^	3.54 (3.09–4.05)^‡^	15.9 (9.89–25.5)^‡^
2019–2021	0.10 (0.09–0.12)^‡^	4.74 (4.14–5.43)^‡^	36.6 (22.9–58.6)^‡^

Results are expressed as odds ratio and 95% confidence intervals (in brackets). *p < 0.05; ^†^*p* < 0.01; ^‡^*p* < 0.001. VTE, venous thromboembolism; DVT, deep vein thrombosis; CrCl, creatinine clearance; Ref., reference. Bold words are the main subjects to study. Years of prescription, number of patients Italic words apply for the total number of patients in each period of study and in the different parts of the world.

### Predictors of use of one direct oral anticoagulant vs. the rest of direct oral anticoagulants

Among patients using DOACs, rivaroxaban was more likely used in men (OR: 1.26; 95% CI: 1.18–1.36) in patients aged <65 years (OR: 1.51; 95% CI: 1.39–1.63) weighing > 120 kg (OR: 1.61; 95% CI: 1.21–2.15) or with prior VTE (OR: 1.23; 95% CI: 1.12–1.36) (Table 4). Apixaban was the preferred DOAC among patients >79 years (OR: 1.59; 95% CI: 1.43–1.76) in those initially presenting with PE (OR: 1.17; 95% CI: 1.08–1.27) with recent bleeding (OR: 1.80; 95% CI: 1.37–2.35) renal insufficiency (OR: 1.89; 95% CI: 1.51–2.38) atrial fibrillation (OR: 1.62; 95% CI: 1.39–1.88) or in fragile patients (OR: 1.79; 95% CI: 1.65–1.94) Edoxaban was much more likely used in Spain than in other countries.

**TABLE 4:** Univariable analyses for predictors of use of each DOAC vs. the rest of DOACs.

	Rivaroxaban		Apixaban		Edoxaban	
			
	*N*	OR (95% CI)	*N*	OR (95% CI)	*N*	OR (95% CI)
***Patients, N***	***6,886***		***3,601***		***1,473***	
**Clinical characteristics**						
Male gender	3,721	1.26 (1.18–1.36)^†^	1,709	0.80 (0.74–0.86)^†^	723	0.90 (0.80–1.00)
Age 65–79 years	2,109	Ref.	1,223	Ref.	502	Ref.
Age < 65 years	3,799	1.51 (1.39–1.63)^‡^	1,415	0.69 (0.63–0.75)^‡^	660	0.85 (0.75–0.96)^†^
Age > 79 years	978	0.64 (0.58–0.71)^‡^	963	1.59 (1.43–1.76)^‡^	311	1.07 (0.92–1.24)
Body weight 50–120 kg	6,646	Ref.	3,478	Ref.	1,439	Ref.
Body weight < 50 kg	96	0.80 (0.60–1.07)	69	1.38 (1.02–1.86)*	22	0.95 (0.61–1.49)
Body weight > 120 kg	143	1.61 (1.21–2.15)^†^	54	0.82 (0.60–1.12)	12	0.43 (0.24–0.78)^†^
**Initial VTE presentation**						
Isolated DVT	2,845	Ref.	1,373	Ref.	672	Ref.
Pulmonary embolism	4,041	0.96 (0.89–1.03)	2,228	1.17 (1.08–1.27)^†^	801	0.80 (0.72–0.89)^†^
**Concomitant disorders**						
Recent major bleeding	103	0.66 (0.51–0.86)^†^	95	1.80 (1.37–2.35)^†^	20	0.72 (0.45–1.14)
CrCl levels < 30 mL/min	122	0.50 (0.40–0.63)^†^	135	1.89 (1.51–2.38)^†^	47	1.32 (0.96–1.81)
Liver cirrhosis	15	1.47 (0.62–3.47)	2	0.23 (0.05–0.98)*	5	2.04 (0.76–5.51)
Platelet count < 100,000/μL	92	0.95 (0.70–1.29)	56	1.21 (0.87–1.67)	15	0.72 (0.42–1.22)
Atrial fibrillation	337	0.57 (0.49–0.66)^†^	308	1.62 (1.39–1.88)^†^	83	0.86 (0.68–1.09)
Prior VTE	1,202	1.23 (1.12–1.36)^†^	569	0.96 (0.86–1.06)	179	0.69 (0.58–0.81)^†^
Fragile patients	1,896	0.55 (0.51–0.59)^†^	1,541	1.79 (1.65–1.94)^†^	538	1.17 (1.04–1.31)^†^
**Countries**						
Spain	1,919	Ref.	1,669	Ref.	873	Ref.
Rest of Europe	3,935	2.52 (2.33–2.73)^‡^	1,509	0.58 (0.53–0.63)^‡^	586	0.45 (0.41–0.51)^‡^
America	569	4.06 (3.42–4.82)^‡^	181	0.55 (0.46–0.66)^‡^	2	0.01 (0.00–0.05)^‡^
Rest of the world	463	2.55 (2.17–3.00)^‡^	242	0.90 (0.76–1.06)	12	0.07 (0.04–0.13)^‡^
**Years**						
2013–2015	2,409	Ref.	305	Ref.	18	Ref.
2016–2018	2,724	0.19 (0.17–0.21)^‡^	1,550	3.70 (3.24–4.23)^‡^	496	17.1 (10.7–27.4)^‡^
2019–2021	1,753	0.10 (0.09–0.11)^‡^	1,746	5.06 (4.43–5.79)^‡^	959	41.1 (25.7–65.6)^‡^

*p < 0.05; ^†^*p* < 0.01; ^‡^*p* < 0.001. VTE, venous thromboembolism; DVT, deep vein thrombosis; CrCl, creatinine clearance; Ref., reference. Bold words are the main subjects to study. Years of prescription, number of patients Italic words apply for the total number of patients in each period of study and in the different parts of the world.

Independent predictors for the use of rivaroxaban (vs. other DOACs) were: male gender (OR: 1.20; 95% CI: 1.10–1.30), age <65 years (OR: 1.47; 95% CI: 1.34–1.61) initial presentation as PE (OR: 1.16; 95% CI: 1.07–1.26) and VTE diagnosis in non-Spanish European countries (OR: 2.17; 95% CI: 1.99–2.36) America (OR: 2.80; 95% CI: 2.32–3.37) or in other countries (OR: 2.67; 95% CI: 2.24–3.18) Rivaroxaban was less prescribed for >79 years (OR: 0.70; 95% CI: 0.62–0.78) recent bleeding (OR: 0.58; 95% CI: 0.43–0.79) and atrial fibrillation (OR: 0.72; 95% CI: 0.61–0.85) (Table 4) Independent predictors for the use of apixaban were: male gender (OR: 0.88; 95% CI: 0.81–0.95) >79 years (OR: 1.46; 95% CI: 1.30–1.64) recent major bleeding (OR: 1.77; 95% CI: 1.33–2.36) atrial fibrillation (OR: 1.22; 95% CI: 1.04–1.44) Apixaban was less used in age <65 years (OR: 0.72; 95% CI: 0.65–0.79) and VTE diagnosis in non-Spanish European countries (OR: 0.67; 95% CI: 0.61–0.73) or in America (OR: 0.82; 95% CI: 0.68–0.98) Independent predictors for the use of edoxaban were: body weight >120 kg (OR: 0.53; 95% CI: 0.29–0.97) initial VTE presentation as PE (OR: 0.64; 95% CI: 0.57–0.72) liver cirrhosis (OR: 3.86; 95% CI: 1.21–12.3) prior VTE (OR: 0.80; 95% CI: 0.67–0.95) and being diagnosed in Spain. Interestingly, the use of rivaroxaban (comparatively with the other two DOACs) progressively decreased over time.

## Discussion

Our findings, obtained from a large cohort of patients with acute VTE in up to 30 countries over the world, reveal large variations in the use of DOACs according to patient factors, institutional factors and also over time. As it could have been expected, the DOACs were more likely used in young patients, those with normal body weight and with no exclusion criteria to be enrolled in the pivotal trials where their indication was based (i.e., recent bleeding, renal insufficiency, liver cirrhosis or thrombocytopenia) Studies about patients preferences usually report more satisfied patients with DOAC than VKA drugs (15, 16) but it seems it is not a reason from prescription in some countries as Spain. Also, its use was much lower in Spain (where the DOACs are not reimbursed) and progressively increased over time. However, there were surprising findings in some subgroups of patients. For example, while the use of DOACs was lower than expected in the subgroups of patients where they had demonstrated to be superior to standard therapy, they were not infrequently used in patients with contraindications to their use.

Subgroup analyses from randomized trials revealed that the DOACs had advantages over standard anticoagulation in fragile patients with VTE. In the EINSTEIN trials, the risk for major bleeding in fragile patients using rivaroxaban was significantly lower than in those on standard therapy (17–19). This difference was not found in non-fragile patients. In the HOKUSAI trial, fragile patients using edoxaban had a significantly higher efficacy than those on VKAs (19). The superiority of the DOACs over standard therapy in fragile patients with VTE was subsequently confirmed in real-life conditions (20, 21). However, only 24% of the 17,767 fragile patients in our cohort used DOACs. We hypothesize that a higher use of DOACs in fragile patients with VTE (40% of the whole series) might have been associated with improved outcomes.

On the other hand, the use of DOACs is contraindicated in patients with severe liver or renal insufficiency, in pregnant or breast-feeding women, and in patients perceived to be at high risk for bleeding (4). Because most of these patients with were excluded from the clinical trials, data regarding their effectiveness and safety are only available through non-randomized studies of which statistical type I/type II errors could play a role (6, 22). Despite this knowledge gap, 23% of patients with recent major bleeding, 15% with CrCl levels <30 mL/min, 11% with cirrhosis, and 16% with thrombocytopenia in our cohort used DOACs. There are few data about resuming anticoagulation after major bleeding with DOAC. In the study from Little (23), reassumption of DOAC in extracranial non-gastrointestinal bleeding was accompanied by reduction in thrombosis. We haven’t studied DOAC in pregnancy in RIETE as opposite to GARFIELD study (24). Data of DOAC used in patient with recently bleeding are an interesting finding since the lack of a monitoring assays say and reversal agents (25) have been important safety concerns for clinicians. A substantial proportion of these patients (25, 44, 26, and 27%, respectively) received lower than recommended doses. This is also of concern, since under-dosing of DOACs has been associated with decreased efficacy and no benefit in safety (26–29). Apixaban was seen to be the most frequent DOAC with dose modification. It could be argued that attending physician has used the same adjustment for dose that has to be made in atrial fibrillation, as it’s supposed in the study from, about changing pattern of type of anticoagulant use and in off-label use a cohort from Switzerland (30). Also low doses are used in atrial fibrillation (31, 32).

Finally, because patients at extremes of body weight were underrepresented in DOAC clinical trials and randomized trials for these patient subgroups are currently unavailable, the International Society of Thrombosis and Hemostasis Scientific and Standardization Committee recently suggested that rivaroxaban and apixaban can be adequate for VTE therapy regardless of body weight, and suggested not using dabigatran, edoxaban or betrixaban in patients weighing >120 kg (12). In our cohort, 12 patients weighing >120 kg used edoxaban (5.6% of the obese patients using DOACs).

Among patients receiving DOACs in our cohort, there was some preference for apixaban over rivaroxaban or edoxaban in the elderly (33) and in patients with recent major bleeding or atrial fibrillation. On the other hand, rivaroxaban was preferred in the young, and edoxaban in those with liver cirrhosis. However, in the absence of clinical trials comparing the DOACs each other, there is no evidence to support that one specific DOAC is superior to any other in terms of efficacy or safety in any clinical scenario.

The main strength of this study is the large size of the RIETE registry, which enabled us to explore the variations across multiple settings, including across patient-related factors, across geographic regions as well over time. As such, the data related to temporal, institutional, and particularly patient-level variations per clinical subgroups provide real-world evidence about contemporary practice and could be helpful for practice management, policy making, and designing future research studies. However, a number of limitations of this study must be acknowledged. First, we did not evaluate the role of patient income and sociodemographic variables as they relate to patient’s willingness to pay for DOACs. Second, factors related to DOACs therapy choice may change over time as prescribers and patients gain more familiarity and experience with the newer DOACs, and additional research will be needed to identify predictors of treatment and changes in DOAC treatment patterns in the future. Third, some countries had fewer participating centers or enrolled only a few patients. As such, although findings from this multicenter, multinational study demonstrate regional variations in diagnostic practices, accurate comparisons for point estimates are not feasible for some countries. Finally, future research needs to consider the impact of patient preferences in DOAC therapy decisions.

This is the first of a series of studies to explore the use of DOACs in patients with VTE and their potential consequences. The focus of the current study was on the assessment and description of potential variations in the choice of drugs for VTE therapy. Future studies are required to explore the reasons behind the variations, the accuracy of each approach, and to assess the impact of these variations on VTE-related and non- VTE-related outcomes in adjusted analyses.

In conclusion, in a large multicenter, multinational registry of patients with VTE, we observed noticeable variations in the choice of DOACs according to the underlying patient factors and institutional factors.

## Data availability statement

The original contributions presented in this study are included in the article further inquiries can be directed to the corresponding author.

## Author contributions

PB, SO, JT, TS, LM, and AB-R: review of draft. All authors contributed to the article, approved the submitted version, and contributed to the patients’ enrollment.

## Abbreviations

DOACs: direct oral anticoagulants
VTE: venous thromboembolism
OR: odds ratio
CI: confidence intervals
LMWH: low-molecular-weight heparin
VKAs: vitamin K antagonists
PE: pulmonary embolism
DVT: deep vein thrombosis
CT-scan: computed tomography scan
CrCl: creatinine clearance.

## Appendix

### Members of the RIETE Group

**SPAIN:** Adarraga MD, Agudo P, Aibar J, Aibar MA, Amado C, Arcelus JI, Ballaz A, Barba R, Barbagelata C, Barrón M, Barrón-Andrés B, Bascuñana J, Blanco-Molina A, Beddar Chaib F, Botella E, Camón AM, Castro J, Chasco L, Criado J, de Ancos C, de Miguel J, del Toro J, Demelo-Rodríguez P, Díaz-Brasero AM, Díaz-Pedroche MC, Díaz-Peromingo JA, Díaz-Simón R, Domínguez IM, Dubois-Silva A, Escribano JC, Espósito F, Farfán-Sedano AI, Fernández-Capitán C, Fernández-Reyes JL, Fidalgo MA, Font C, Francisco I, Gabara C, Galeano-Valle F, García MA, García-Bragado F, García de Herreros M, García de la Garza R, García-Díaz C, García-Mullor MM, Gil-Díaz A, Gómez-Cuervo C, Gómez-Mosquera AM, González-Martínez J, Grau E, Guirado L, Gutiérrez J, Hernández-Blasco L, Jara-Palomares L, Jaras MJ, Jiménez D, Jiménez R, Jiménez-Alfaro C, Jou I, Joya MD, Lainez-Justo S, Latorre-Díez A, Lalueza A, Lecumberri R, Lobo JL, López-Brull H, López-De la Fuente M, López-Jiménez L, López-Miguel P, López-Núñez JJ, López-Reyes R, López-Sáez JB, Lorenzo A, Lumbierres M, Madridano O, Maestre A, Marchena PJ, Marcos M, Martín-Martos F, Martínez-Urbistondo D, Mella C, Mellado M, Mercado MI, Monreal M, Muñoz-Blanco A, Muñoz-Gamito G, Morales MV, Nieto JA, Núñez-Fernández MJ, Olid-Velilla M, Otalora S, Otero R, Parra P, Parra V, Pedrajas JM, Pellejero G, Peris ML, Porras JA, Portillo J, Rivera A, Roca M, Rosa V, Ruiz-Artacho P, Ruiz-Giménez N, Ruiz-Ruiz J, Ruiz-Sada P, Salgueiro G, Sánchez-Muñoz-Torrero JF, Sancho T, Sigüenza P, Soler S, Suriñach JM, Torres MI, Trujillo-Santos J, Uresandi F, Usandizaga E, Valle R, Varona JF, Vela L, Vela JR, Villalobos A, Villares P, Zamora C, **AUSTRIA:** Ay C, Nopp S, Pabinger I, **BELGIUM:** Engelen MM, Vanassche T, Verhamme P, **COLOMBIA:** Esguerra G, Montenegro AC, Roa J, **CZECH REPUBLIC:** Hirmerova J, Malý R, **FRANCE:** Accassat S, Bertoletti L, Bura-Riviere A, Catella J, Chopard R, Couturaud F, Espitia O, El Harake S, Helfer H, Le Mao R, Mahé I, Moustafa F, Poenou G, Sarlon-Bartoli G, Suchon P, **GERMANY:** Schellong S, **ISRAEL:** Braester A, Brenner B, Kenet G, Tzoran I, **ITALY:** Basaglia M, Bilora F, Bortoluzzi C, Brandolin B, Ciammaichella M, Colaizzo D, De Angelis A, Di Micco P, Grandone E, Imbalzano E, Mastroiacovo D, Merla S, Pesavento R, Prandoni P, Siniscalchi C, Tufano A, Visonà A, Vo Hong N, Zalunardo B, **LATVIA:** Kalejs RV, Rusa E, Skride A, **PORTUGAL:** Fonseca S, Manuel M, Meireles J, **REPUBLIC OF MACEDONIA:** Bosevski M, Krstevski G, **SWITZERLAND:** Bounameaux H, Mazzolai L, **USA:** Caprini JA, Weinberg I, **VIETNAM:** Bui HM.

**Coordinator of the RIETE Registry:** Manuel Monreal.

**RIETE Steering Committee Members:** Paolo Prandoni, Benjamin Brenner, and Dominique Farge-Bancel.

**RIETE National Coordinators:** Raquel Barba (Spain), Pierpaolo Di Micco (Italy), Laurent Bertoletti (France), Sebastian Schellong (Germany), Inna Tzoran (Israel), Abilio Reis (Portugal), Marijan Bosevski (R. Macedonia), Henri Bounameaux (Switzerland), Radovan Malý (Czech Republic), Peter Verhamme (Belgium), Joseph A. Caprini (USA), Hanh My Bui (Vietnam).

**RIETE Registry Coordinating Center:** S&H Medical Science Service.
